# Pinch force sense test–retest reliability evaluation using contralateral force matching task

**DOI:** 10.1038/s41598-024-51644-0

**Published:** 2024-01-11

**Authors:** Lin Li, Shuwang Li

**Affiliations:** Department of Physical Education, Renmin University of China, No. 59 Zhongguancun Street, Beijing, 100872 China

**Keywords:** Motor control, Somatosensory system

## Abstract

A high test–retest reliability in measurement of pinch force sense is required to assess a clinical parameter accurately over a longitudinal study. Ipsilateral reproduction (IR) task and contralateral matching (CM) task have commonly been used for the assessment of force sense. To date, there has been little research on the test–retest reliability of pinch force sense utilizing the contralateral force matching task. This research aimed to explore this phenomenon across a spectrum of reference force levels (10, 30, and 50 percent maximum voluntary isometric contraction (MVIC)) using a contralateral matching task. Every participant in the study was tested twice by the same skilled experts, with each session separated by one week. Although normalized variable error indicated a poor level of reliability (intraclass correlation coefficient (ICC) = − 0.25 to 0.05) for these force sense tests, normalized constant error (ICC = 0.76–0.85) and normalized absolute error (ICC = 0.61–0.81) results indicated a fair to good of reliability. The lower bound of 95% CI of ICC for NAE and NCE indicated fair test–retest reliability (0.41–0.69). These findings suggest that investigators can reasonably obtain a fair to good test–retest reliability when investigating pinch force sense using the contralateral matching task. The Bland–Altman plots, SEM, and MDD95% were lower at these lower reference force level (10% MVIC) compared to the level of higher reference forces (30% and 50% MVIC). Therefore, when the reference force level increases, the participant needs a larger NAE or NCE decrease to show that their pinch force sense has indeed improved.

## Introduction

Proprioception is a sensation that is necessary for proper and concise mobility^1,2^. Force sense, joint position sense, and kinesthesia are the 3 basic conscious proprioceptive senses essential to optimal neuromuscular regulation, with force sense particularly crucial in this case^3,4^. The individual capability to correctly recognize and understand external or internal forces connected with a certain joint is called force sense^5,6^.

People need the ability to self-monitor and control their pinch force to do tasks with competence, such as rock climbing and writing with a pen^7^. The capacity to impulsively pinch a particular object is a complex motor activity requiring adequate power to guarantee that the object remains in position while avoiding excessive force that could cause damage to the object or hand exhaustion. Persistent and unnecessary high pinch forces are considered risk factors for musculoskeletal disorders (MSDs), including carpal tunnel syndrome^8–10^, tendonitis^11^, and epicondylitis^12^. Recurrent pinch forces in the workplace may be more robust, which is crucial for MSDs since most injuries result from chronic slight distresses. Repetitive and excessively high pinch forces may induce mild and chronic strain on a few structures such as bones, joints, muscles, tendons, etc., increasing the likelihood of catastrophic damage^13^. Understanding the force sense of pinch is vital to understanding the reasons for common MSDs and designing remedies to lower injury risks.

Different studies have used various force sense tests to examine pinch force perception^14,15^. Ipsilateral reproduction (IR) task^16^ and contralateral matching (CM) task^17^ have commonly been used for the assessment of force sense. Participants had recreated the previously experienced target force using the same (ipsilateral) hand in the IR task. The task of contralateral matching requires corresponding an equal force with the opposite hand. In this scenario, the force set before matching stays at the reference force and can be used as an "online" reference to help match participants. Force sense is measured by how well forces are matched (reproduced).

Choosing between ipsilateral and contralateral reproduction (matching) tasks may appear simple. However, this is not the case. For instance, in IR, where the same hand is used to establish both the target and reproducing force, participants must rely on memory to successfully duplicate the target force. Considering the memory aspect of this sort of activity, IR should be used with caution when assessing proprioceptive acuity in persons who are prone to memory problems. In this case, some of the reproduction error recorded is due to cognitive or memory deficiencies^18^ rather than a loss in proprioception. Because the opposite hand makes the reference force available throughout the task, using CM reduces the necessity for memory-based matching. Although this benefit, CM were subject to their restrictions. Based on the physical pathways associated with transmitting peripheral proprioceptive input to the brain, matching with the opposite hand necessitates more interhemispheric transfer than IR. This crossover most likely occurs via the corpus callosum's transcallosal pathways^19–21^.

Researchers need to be conscious of the benefits and limitations of each method of the force sense. Another essential factor when selecting between ipsilateral and contralateral reproduction (matching) tasks is test–retest reliability. A high test–retest reliability in a specific tool or technique is required to assess a clinical parameter accurately over a longitudinal study. If a test is credible, a clinician may make reliable results that are not significantly influenced by external influences, minimizing the chances of an incorrect conclusion. Earlier research has stated that the test–retest reliability of force sense in the ankles^22^, knees^23^, hips^24^, and shoulders^25^ indicate different reliability across joints for these tests. A study also reported the test–retest reproducibility of IR-based pinch force tests. On the basis of the high intraclass correlation coefficient values (0.783–0.895), the ipsilateral pinch force reproduction task revealed good reliability at 10%, 30%, and 50% MVIC^26^.

However, reliability in the contralateral pinch force matching task-related results has not been evaluated. By comparing different force levels, force reproduction (matching) error does not appear to be a linear process^27–30^, and the pinch grip coordination difficulty increases with the force level^31,32^. Therefore, force levels may be expected to interact with the test–retest reliability. As a result, the present research aimed to explore the test–retest reliability for the contralateral pinch force matching task at various force levels.

## Materials and methods

### Study participants

A total of 30 healthy participants (15 women and 15 men, age 20.4 ± 2.7 years, weight 65.0 ± 12.2 kg, height 172.5 ± 8.1 cm, hand length 18.1 ± 1.0 cm, all right-handed) participated in the present study. The sample size was determined by reference to a statement by Fleiss that a group of 15–20 is adequate for assessing the stability of a quantitative variable^33^. The handedness of all participants was assessed using the Edinburgh Handedness Inventory^34^. The laterality quotient was larger or equal to 60 (Laterality Quotient: 92.1 ± 10.9). The participants presented no neuromuscular disorders and were naive to the task. Informed consent form was obtained from all of the participants. The ethical review board of Renmin University of China (reference number 2022177) approved the present research/experiment. This study was performed in conformity with the principles of the Declaration of Helsinki.

### Apparatus

**Figure 1:**
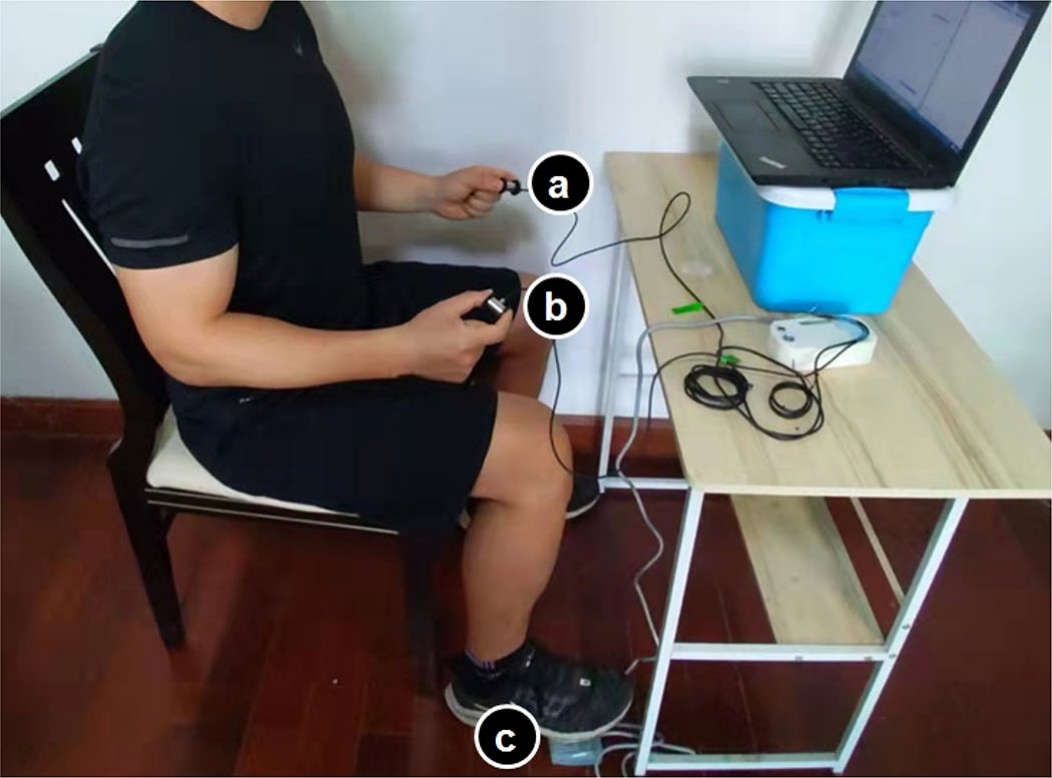
The standard testing position was utilized in all pinch force sensitivity tests. Each participant in the trial sat on a chair that was 60 cm from the screen. (**a**,**b**) The subject holds two electronic digital force dynamometers in both hands and (**c**) a footswitch under his right foot.

All strength and force matching tests were done with two electronic digital force dynamometers (Pinch analyzer, China, range of measurement: 0–100 N, accuracy: 0.05 N; Kjyl Technologies, Beijing, China) of the same size and shape. The two electronic digital force dynamometers (Fig. 1a,b) were used to measure the right and left pinch force, respectively. The calibration configuration from the manufacturer was used, and the instrument was checked out before the laboratory activity. The devices had a pinch span of 2.5 cm, and the sampling rate stayed at 100 Hz. The outputs from the electronic digital force dynamometer were converted and amplified by a A/D Converters (AD7190,ADInstruments, Bella Vista, NSW, Australia) and low-pass filtered at 5 Hz (Butterworth, fourth-order, zero-phase lag). A protocol was made to measure pinch force sense. It was based on the pinch analyzer.

### Protocol

Auditory disruptions were reduced by performing the study in a quiet area^35,36^. The respondents sat in a chair about 60 cm in front of a computer monitor. They then put their whole bodies in a line as per the American Society of Hand Therapists guidelines (Fig. 1): their upper arms were vertical, their elbows were bent at 90°, and their forearm and wrists were in a neutral position^37^. For the tip pinch, the tip of the thumb is moved to the tip of the index finger, and the other fingers are bent entirely^38^. The respondents were advised to hold this position throughout the test, and their right-hand force output was displayed on the computer screen. A customized maximum voluntary isometric contraction (MVIC) test software and a contralateral force matching task software (Kjyl Technologies, Beijing, China) were used to capture and analyze data on a computer.

#### The MVIC test

The participants engaged in warm-up activities before testing. They were instructed to use either their right or left hand (randomly) and apply the maximum pinch force to the dynamometer for 5 s, reach this maximum within 1 s, maintain it for 3 s, and relax within 1 s. The pinch strength was determined by doing the test three times. The average value of three trials of maximal value each trial was recorded as the pinch strength. Participants were allowed to rest for 2 min between tests to lessen the impact of fatigue on research findings.

#### Contralateral force matching task

**Figure 2:**
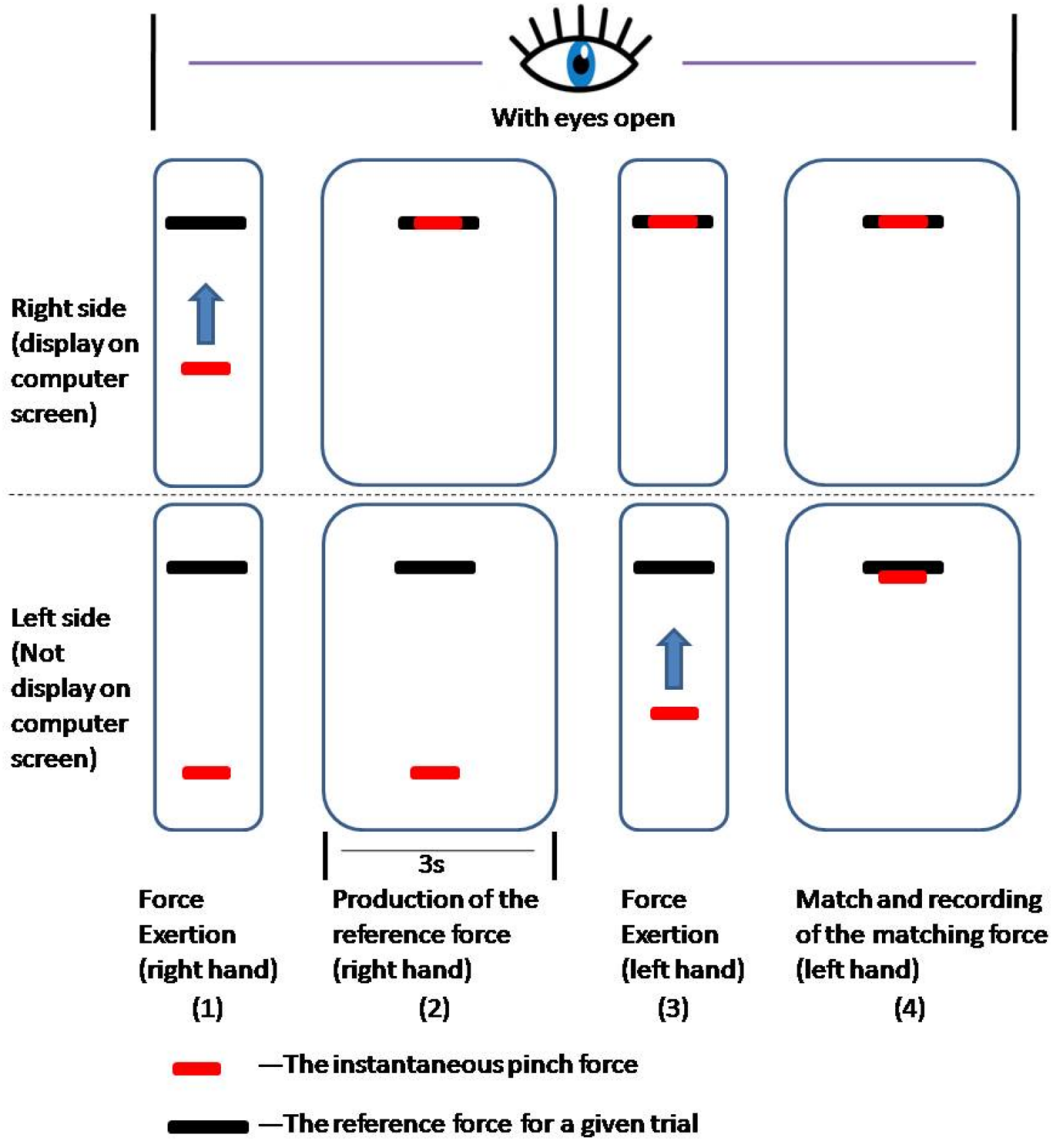
A simplified version of the user interface used to help guide study subjects to a comfortable degree of force. When the respondents opened their eyes, the red line representing pinch force in the (1) right hand was at the bottom and had been elevated to overlap with the black line above. (2) After three seconds, the participants must align the red line representing pinch force in the (3) left hand as closely as possible with the black line. (4) The black lines represent reference force, whereas the red lines represent instantaneous pinch force.

**Figure 3:**
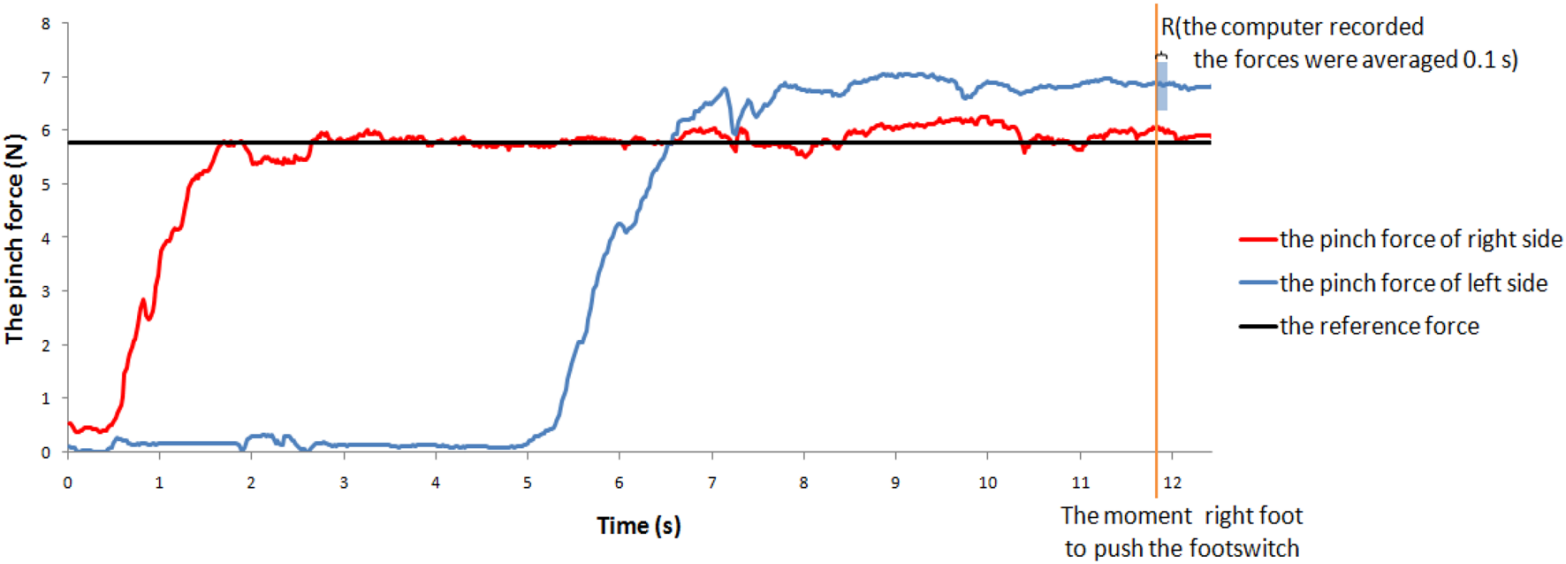
A raw force data of an exemplar participant performing the contralateral force matching task.

Participants' ability to precisely match a reference force was used to evaluate force perception. Initially, participants viewed a video explaining the task. Then, using a bespoke C^++^ application, participants were shown a black line representing the reference force in a specific trial. A red line on the same display indicated the instant pinch force (Fig. 2). The respondents were asked to exert a reference force with their right hand, T, for 3 s, and the reference (right) hand was held at the reference force while the opposite (left) hand actively matched the force. Participants were asked to use their right foot to push the footswitch (Fig. 1c) when they believed they had successfully reached the reference force, following which the computer recorded the forces were averaged over 0.1 s (R, in the left hand) (Fig. 3). Each respondent was then instructed to relax to ensure the participants were comfortable with the procedures. This was done three times during the pre-experiment period. Then, participants were exposed to one of three reference force levels (10, 30, or 50% MVIC) in a randomized order, with three repetitions of each force level. 30 participants for each level. Participants were given a 30-s break between trials, with a longer 2–3-min gap between every 5 trials to mitigate tiredness and maintain mental acuity^39^.

**Figure 4:**
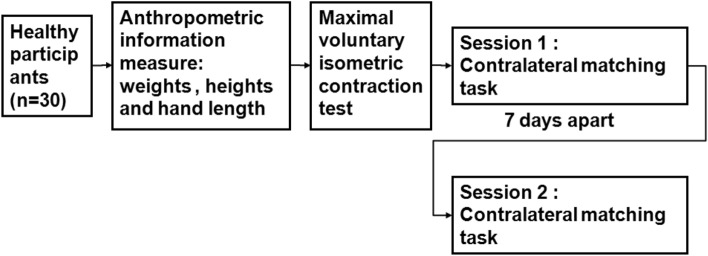
shows a flow chart of the trial carried out. According to the experiment's flowchart, 30 healthy subjects measured their hand length, weight, and height. The maximum voluntary isometric contraction was tested. Thirdly, the contralateral force matching task was evaluated. The contralateral force matching task was repeated after 7 days.

**Figure 5:**
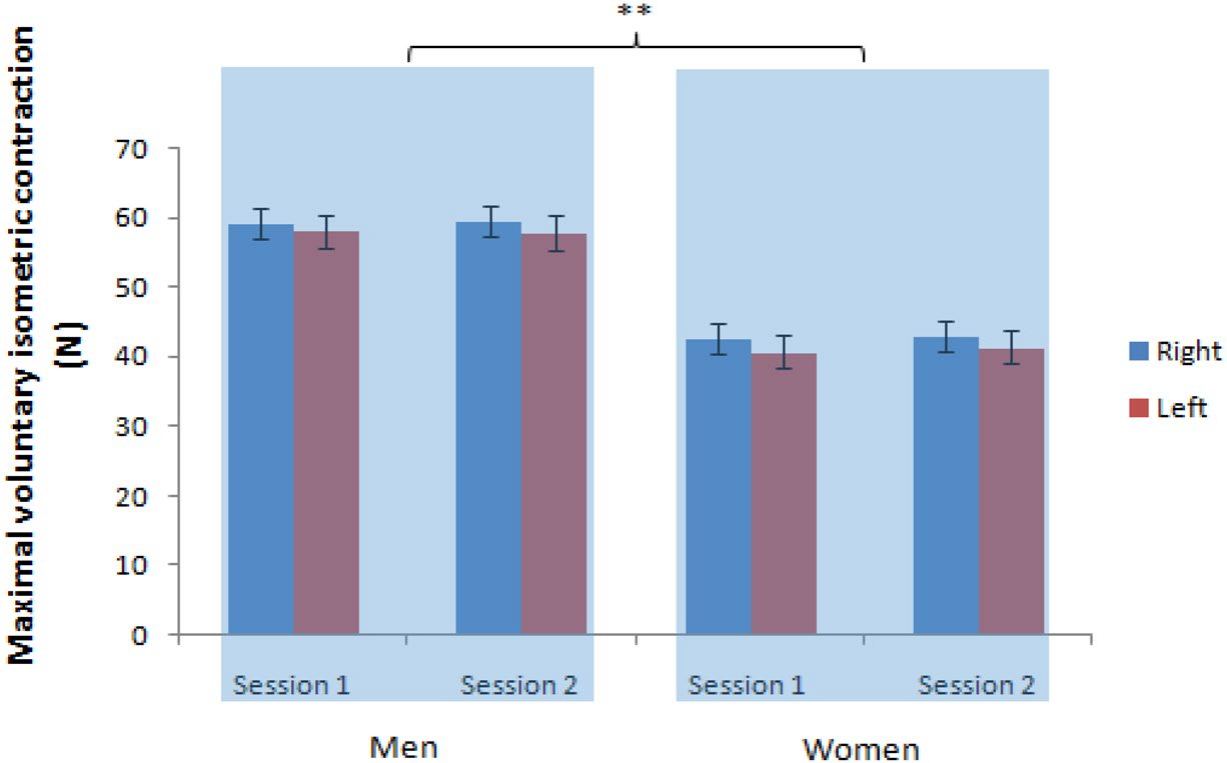
The maximum voluntary isometric contraction as a function of the sex, hands and sessions (** = P < 0.01).

Pinch force sensitivity was assessed by having subjects perform 9 trials throughout two sessions (Session 1 and Session 2). Each session occurred around 7 days apart and was conducted in the same lab by the same investigator^39,40^. The random order in which experiments were replicated over the two 9-trial sessions differed. All respondents described that, between sessions, they had not developed any functional or health-related issues with the potential to disrupt study performance. Individuals were also instructed to refrain from participating in any physical activity shortly before the actual test or between the test and retest session to decrease the possibility of fatigue influencing the research findings (Fig. 4). Visual task-related signals were displayed to study participants using proprietary C^++^ software (Fig. 5).

### Statistical analyses

The variable error (VE), constant error (CE), and absolute error (AE) were used to assess force perception errors. The overall errors are described by AE, while the directionality of errors is described by CE, and the variability of errors over trials is represented by VE to indicate the accuracy with which the performances were achieved. To consider force differences between individuals that simply arise due to differences in body mass (BM), AE, CE, and VE were calculated with the error normalized to BM to obtain the normalized absolute error (NAE), normalized constant error (NCE), and normalized variable error (NVE), respectively. The equations are as follows:

1 $$NAE=\frac{{\sum }_{i=1}^{3}\left|{R}_{i}-T\right|}{3\cdot BM}\times 100\text{\%},\left(i=\mathrm{1,2},3\right),$$

2 $$NCE=\frac{{\sum }_{i=1}^{3}\left({R}_{i}-T\right)}{3\cdot BM}\times 100\text{\%},\left(i=\mathrm{1,2},3\right),$$

3 $$NVE=\sqrt{\frac{{\sum }_{i=1}^{3}{\left({R}_{i}-\overline{R }\right)}^{2}}{3\cdot BM}}\times 100\text{\%},\left(i=\mathrm{1,2},3\right)$$

*R*_*i*_ denotes the matching force in the *i-th* trial, *T* the reference force, *BM* the body mass, and $\overline{R}$ the mean matched force across triplicate experiments.

For this purpose, we used the Kolmogorov–Smirnov test to evaluate if the distributions of the study's data were normal. Mixed-model ANOVAs (2-way ANOVA with repeated measures) were used to assess the impact of the hands (right- and left-hand), sessions (session 1 and session 2) and sex (men or women) on MVIC; in this model, sex was selected to be the between subject factor, whereas hands and sessions to be the within-subject factor. Simple-effects analyses were conducted when interaction is observed, and main effects without interaction were compared via a post hoc least significant difference (LSD) test. There is debate over the most appropriate methods for determining reliability and measurement error^41^, with some arguing that no single method is sufficient^42,43^. Consequently, the test–retest reliability was evaluated using a variety of statistical techniques, as given below: (1) MD (mean difference)^40^ and (2) ICC (intraclass correlation coefficient)^44^. Statistics were determined using two-way mixed effects, absolute agreement, and single-measurement models^45^. ICC values of 0.00–0.39 were poor, values of 0.40–0.74 were fair, and values of 0.75–1.00 were good^46^. (3) We calculated the standard measurement error (SEM) to obtain the absolute index of reliability^43^, which was expressed in actual units and unaffected by participant differences. Higher SEM values correspond to greater error levels, indicating that the results tested cannot be replicated. (4) Minimal detectable difference (MDD) 95% represents the smallest difference within a 95% confidence interval that can be identified as the true change in pinch force sense^47,48^. (5) A scatterplot depicting variations between sessions plotted against the mean values with 95% limits of agreement (LOA) (LOA = mean difference ± 1.96 SD) was created using the Bland and Altman method to measure agreement among individual study subjects^49,50^. The data are reported as means standard deviations (± SD), with P < 0.05 set as the significant level. Data were analyzed using SPSS 22.0 (IBM, Armonk, NY, USA).

## Results

Mixed-model ANOVA was used to compute the MVIC, revealing no significant interaction between the sex, hands and sessions, F(1, 29) = 3.94, P = 0.057, the sex and hands, F(1, 29) = 0.01, P = 0.909, the sex and sessions, F(1, 29) = 1.57, P = 0.221, and hands and sessions, F(1, 29) = 0.34, P = 0.563. The MVIC between the sex [men: 58.7 ± 2.1 N and women: 41.9 ± 2.1 N, F(1, 29) = 30.66, P < 0.001] was significantly different, but not hands [right hand: 51.1 ± 1.6 N and left hand: 49.5 ± 1.7 N, F(1, 29) = 1.14, P = 0.296] and sessions [sessions 1: 50.2 ± 1.5 N and sessions 2: 50.4 ± 1.5 N, F(1, 29) = 1.78, P = 0.193].

**Figure 6:**
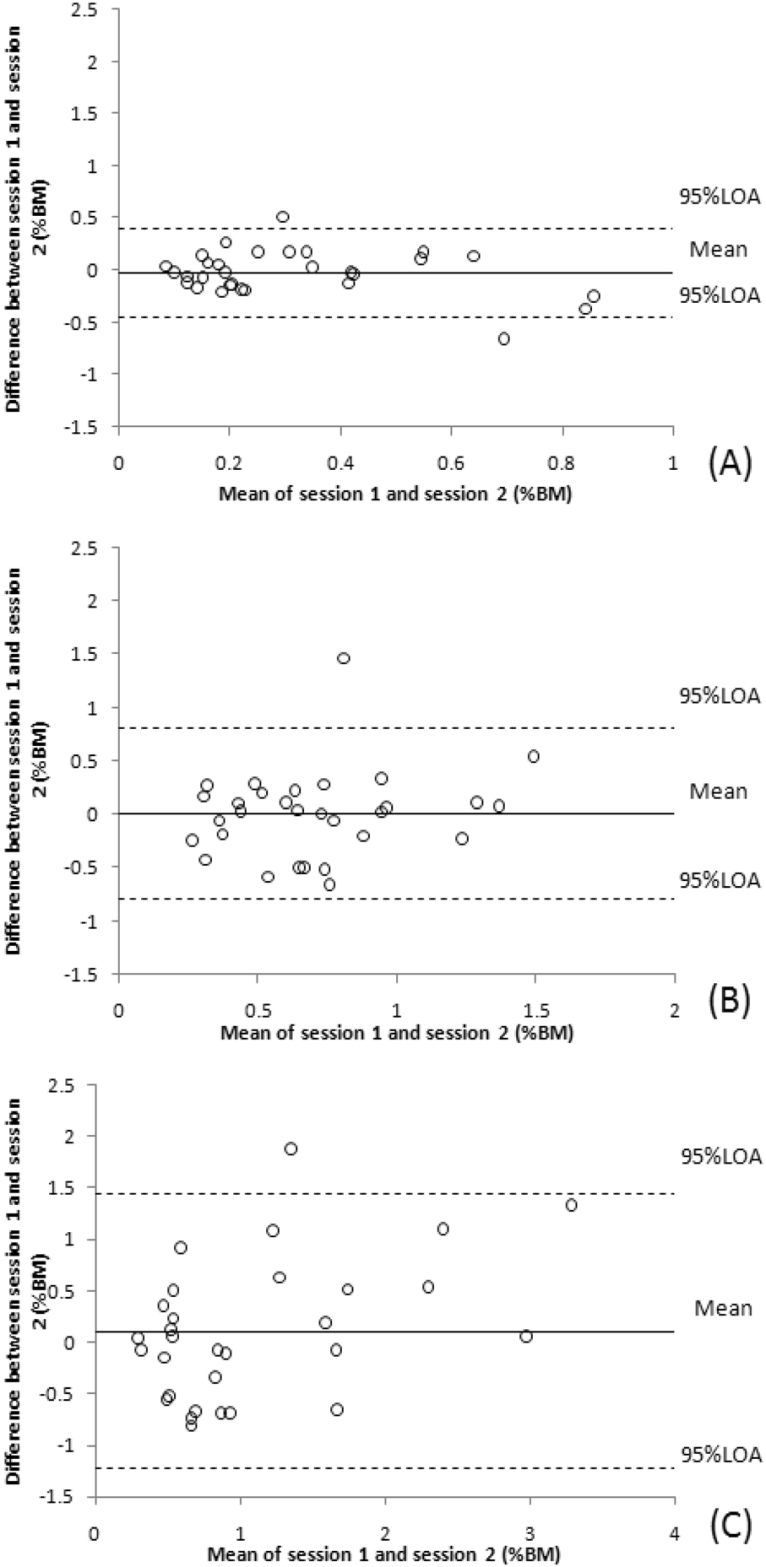
Demonstrates Bland–Altman graphs illustrating the association between the mean test and retest NAE in the differences of retest–test mean NAE at 10, 30, and 50% MVIC reference force levels. The ordinates axis was drawn given the disparity between two parameters, while the abscissa axis was drawn based on the mean of two measurements. The top and lower large-dashed lines represent the 95% limits of agreement (LOA), whereas the solid center line represents the average difference between sessions.

**Figure 7:**
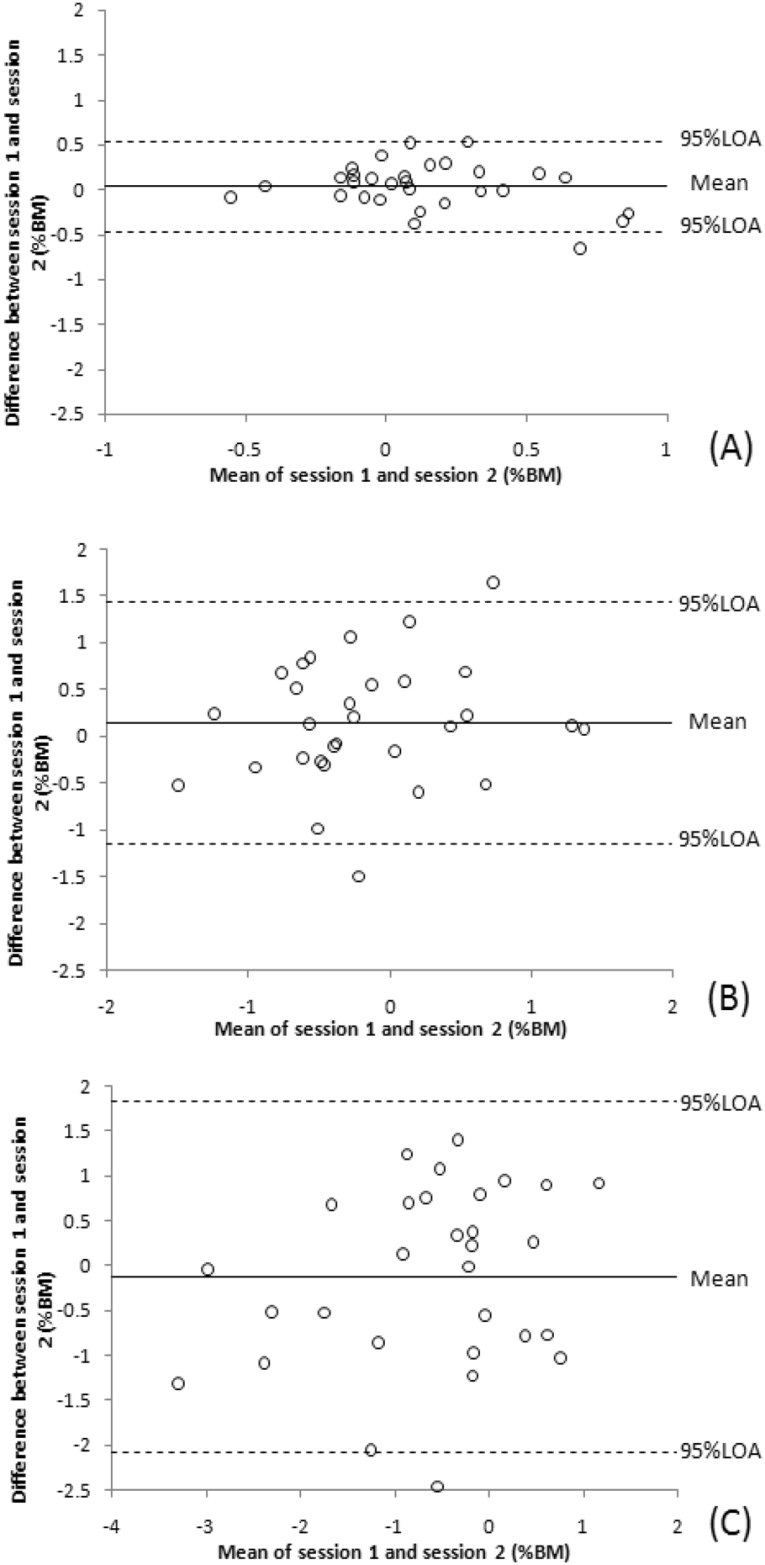
Demonstrates Bland–Altman graphs illustrating the association between the mean test and retest NCE in the differences of retest–test mean NCE at 10, 30, and 50% MVIC reference force levels. The ordinates axis was drawn given the disparity between two parameters, while the abscissa axis was drawn based on the mean of two measurements. The top and lower large-dashed lines represent the 95% limits of agreement (LOA), whereas the solid center line represents the average difference between sessions.

**Figure 8:**
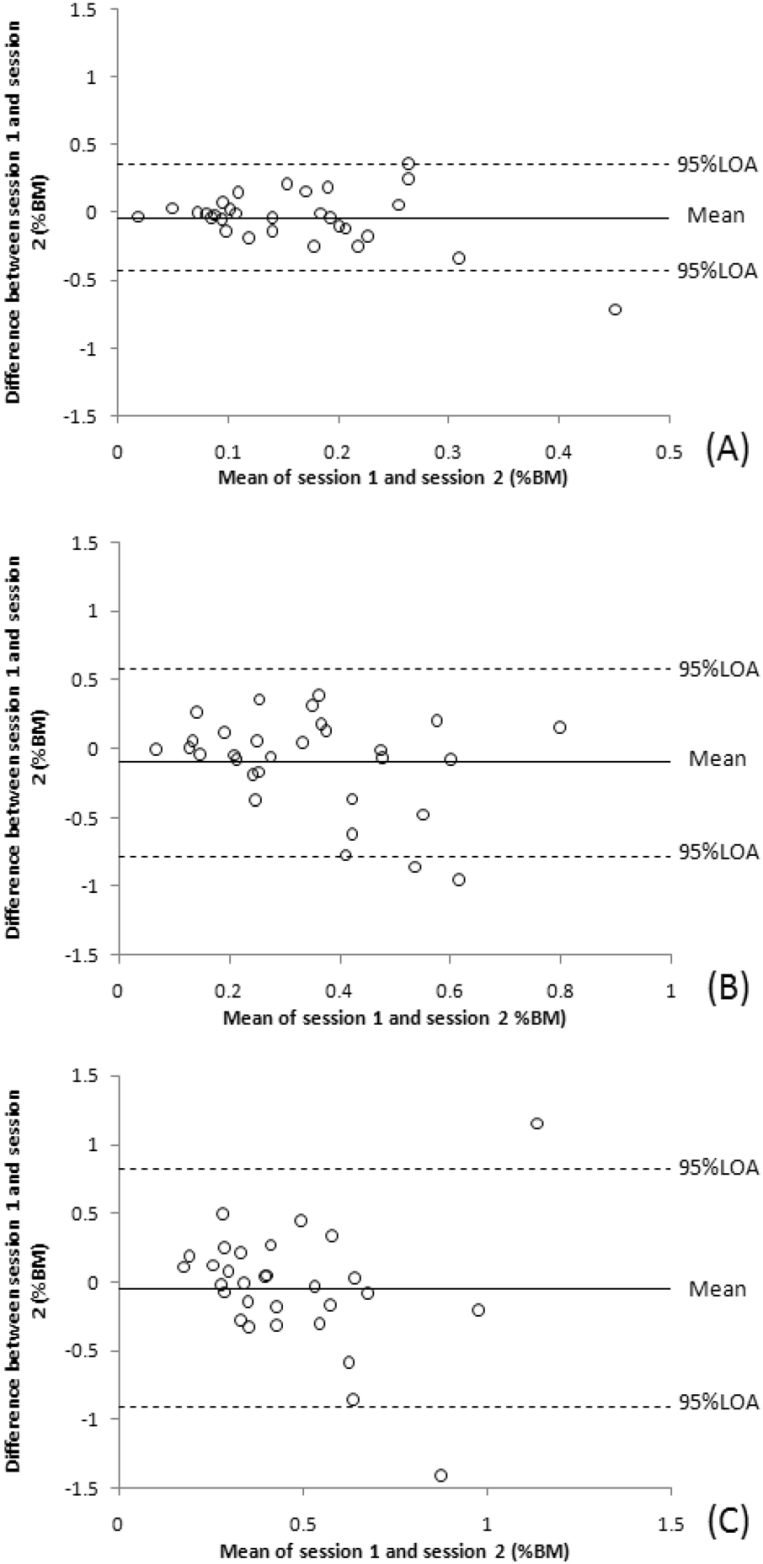
Demonstrates Bland–Altman graphs illustrating the association between the mean test and retest NVE in the differences of retest–test mean NVE at 10, 30, and 50% MVIC reference force levels. The ordinates axis was drawn given the disparity between two parameters, while the abscissa axis was drawn based on the mean of two measurements. The top and lower large-dashed lines represent the 95% limits of agreement (LOA), whereas the solid center line represents the average difference between sessions.

Table 1 shows the estimated NVE, NCE and NAE values for test–retest reliability measurements at 3 reference pinch force levels and the ICC, SEM, MDD95%, and 95% LOA values from the statistical analyses. For these diverse force levels, the NCE (0.76 to 0.85) and NAE (0.61 to 0.81) ICC values indicated fair to good test–retest reliability; however, the ICCs for NVE (-0.25 to 0.05) indicated poor test–retest reliability. The lower bound of 95% CI of ICC for NAE and NCE indicated fair test–retest reliability (0.41–0.69). SEM and MDD95% values were lower at the 10%MVIC reference force levels than at the 30% and 50% MVIC reference force level. Figures 6, 7 and 8 demonstrate Bland–Altman graphs conforming to these calculations. Bland–Altman plots are used to evaluate the agreement amongst research participants. A scatterplot was created to illustrate the differences in the mean absolute error values and LOA at 95% between the sessions. The most significant instances occurred between the two different dashed lines.

**Table 1:** shows the test–retest reliability of pinch force sense.

Force level (% MVIC)	Session 1 (%BM)	Session 2 (%BM)	Mean difference (%BM)	ICC (95% CI, *p* values)	SEM (%BM)	MDD95%(%BM)	95% LOA (%BM)
NAE							
10	0.33 ± 0.69	0.31 ± 0.21	− 0.03 ± 0.22	0.75 (0.48–0.88, < 0.001)	0.11	0.30	− 0.45–0.40
30	0.71 ± 1.31	0.71 ± 0.27	0.00 ± 0.41	0.61 (0.41–0.82, 0.005)	0.25	0.70	− 0.80–0.81
50	1.05 ± 1.08	1.16 ± 0.42	0.11 ± 0.68	0.81 (0.61–0.91, < 0.001)	0.29	0.81	− 1.22–1.44
NCE							
10	0.13 ± 0.10	0.16 ± 0.35	0.03 ± 0.26	0.85 (0.69–0.93, < 0.001)	0.10	0.27	− 0.47–0.54
30	− 0.23 ± 0.16	− 0.09 ± 0.98	0.14 ± 0.66	0.76 (0.49–0.88, < 0.001)	0.32	0.90	− 1.15–1.43
50	− 0.56 ± 0.20	− 0.68 ± 0.71	− 0.12 ± 1.00	0.79 (0.57–0.90, < 0.001)	0.45	1.26	− 2.08–1.83
NVE							
10	0.18 ± 0.28	0.14 ± 0.32	− 0.04 ± 0.20	− 0.25 (− 1.6 to0.41, 0.526)	0.22	0.62	− 0.43–0.35
30	0.40 ± 0.30	0.30 ± 0.39	− 0.10 ± 0.35	0.02 (− 0.95 to 0.52, 0.948)	0.34	0.95	− 0.78–0.58
50	0.50 ± 0.33	0.45 ± 0.79	− 0.05 ± 0.44	0.05 (− 1.05 to 0.55, 0.898)	0.43	1.20	− 0.91–0.82

At triplicate tested pinch force levels, the data correspond to 95% limits of agreement (LOA), standard error of measurement (SEM), minimal detectable difference 95% (MDD95%), intraclass correlation coefficient (ICC) with p values, mean normalized absolute error (NAE), normalized variable error (NVE), normalized constant error (NCE), and between test and retest values.

## Discussion

### The reliability of test–retest

Our results revealed that the test–retest reliability of pinch force sense using the CM task was fair to good (AE: ICC = 0.61–0.81, NCE: ICC = 0.76–0.85), but poor reliable for NVE ( ICC = − 0.25 to 0.05). The lower bound of 95% CI of ICC for NAE and NCE indicated fair test–retest reliability (0.41–0.69). Benjaminse et al. concluded in a previous study that while hip joint force perception during flexion exhibited good test–retest reliability (ICC = 0.764), assessments of hip force perception in other planes were inadequate^24^. ICCs for VE and AE measured in the ankle joint force sense studies were greater than 0.75 despite being lower than 0.75 when evaluating CE^22^. Force sense measurements for AE and CE have been shown to be fair reliable (AE: ICC = 0.42–0.63, CE: ICC = 0.49–0.60), but poor reliable for VE ( ICC = − 0.85 to 0.14) in studies of the hand grip^51^. Furthermore, investigations on the shoulder (internal rotation: ICC = 0.981, exterior rotation: ICC = 0.978)^25^ and the knee (ICC = 0.73–0.81) have revealed that force sense assessments are extremely accurate^23^. In addition, a recent study demonstrated that the test–retest reliability of pinch force sense using the IR task was excellent (AE: ICC = 0.78–0.88, CE: ICC = 0.86–0.90)^26^.

Based on a review of the relevant literature, no one has ever used contralateral force matching tests to evaluate how reliable pinch force sensation is. Our existing data demonstrated that the test–retest reliability of the pinch force sensation in healthy individuals can generate stable and similar measurements, with comparable NAE and NCE readings at three reference force levels (10, 30, and 50% MVIC). When measuring pinch force sensitivity using the contralateral force matching task, the fair to good levels of reliability related with these two types of error were considered acceptable. In contrast, the ICC for VE was of poor reliability. Absolute reliability or compliance was also assessed to investigate the degree of the measurement error^52^, with SEM and MDD95% allowing assessment of the absolute index of reliability in terms of the measuring units. When compared to the 30% and 50% MVIC reference force, the SEM and MDD95% values for the 10% MVIC reference forces were lower.

Bland–Altman plots revealed that one value (3.3%) fell outside the 95% LOA range in Figs. 6C,B, 7C and 8A whereas two (6.7%) values fell beyond this range in Figs. 6A, 7A,B and 8B, C, respectively. Compared to the ranges for 95% LOA at 30% and 50% MVIC, those at 10% MVIC were smaller. Consistent with this observation. The Bland–Altman plots, SEM, and MDD95% were lower at these lower reference force level (10% MVIC) compared to the level of higher reference forces (30% and 50% MVIC). MDD95% refers to the minimum value that can be determined as the true change in the measured data. For example, the MDD95% value of 10%MVIC reference force for NAE obtained from repeated measurements is 0.3% BM. This means that only when the NAE decreases by more than 0.3% BM can we conclude that the participant's pinch force sense has indeed improved. Therefore, as the reference force level increases, the participant would need a larger decrease in NAE or NCE to show an improvement in their pinch force sense.

### The reliability influencing factors

The overall reliability of an experiment can be affected by several factors, such as the effect of attention, learning, and fatigue, which vary between the first test and retest sessions. Rest intervals of 30 s between trials and 2–3 min after completing 5 tests were provided to participants in this study to prevent fatigue^39^.

There were no significant differences between Sessions 1 and 2 in terms of NAE, NCE, or NVE, suggesting that learning did not affect the outcomes of the second session. In order to minimize the possibility of a learning effect, participants were permitted to perform the force matching task until they were comfortable performing these estimates and that they understood all the instructions. The ability to reliably reproduce test results can also be improved through familiarization^21,53–55^. To further mitigate the impact of test–retest time disparities on learning, we mixed up the order in which participants were exposed to reference pressures. This study also included a seven-day interval between testing sessions to mitigate the effects of fatigue and retention on the study's findings^24,40,56,57^. CM requires the concurrent evaluation of two pinch forces and therefore requires more concentration. The present process was carried out in a peaceful setting to lessen the likelihood of distractions.

### Limitations

This study used a standardized technique for measuring pinch force perception to provide consistent experimental circumstances between testing sessions, thereby reducing the potential for differences in the conditions for the test and retest measurements. However, there are restrictions on how far one can go with this approach. The first limitation was the study participants' average young age (± 20.4 years). So, these results may only be accurate when tested on healthy people the same age who have the same pinch force sense. Additional investigation is necessary to apply these outcomes to other age and gender categories. We lowered but did not investigate the effect of trial order (such as interhemispheric transfer, attention, learning, and fatigue). At the same time, we plan to explore it more in subsequent investigations. Follow-up research should be conducted to determine whether parameters such as reference force levels, trial number, or multiple raters are relevant pinch variables affecting pinch sense reliability in normal and diseased individuals.

### Practical implications

Various hand tools and equipment, such as pen, scalpel, or tweezers can exert significant forces and control the object during many production activities. As a substantial aspect of hand tool design, pinch force sensation can reduce hand muscle fatigue and the danger of developing MSDs on the hand^13^. Therefore, analyzing pinch force sensation is critical for optimizing hand tool and device designs. Constant procedures requiring less precision and regularity may initially place the hands under minor strain, but with time they will sustain significant injuries^58^. The evaluation of pinch force sense could enhance understanding of the causes of MSD and lead to developing interventions to reduce injury risks. In addition, pinch force sense could be used to evaluate wounded workers and determine when they can return to work.

## Conclusion

In conclusion, this investigation confirms that assessments of pinch force sensation in healthy adults employing a contralateral force matching task are reasonably accurate. We have found that reference force levels of 10% MVIC were related to lower SEM, MDD95%, and smaller 95% LOA for absolute and constant error values compared to 30% and 50% MVIC. This indicates that a larger decrease in NAE or NCE is required for the participant to demonstrate improved pinch force sense as the reference force level increases.

## Data Availability

The data and materials for all experiments are available at https://osf.io/sqmyk/, and none of the experiments was preregistered.
